# Long-Term Monitoring of Field Trial Sites with Genetically Modified Oilseed Rape (*Brassica napus* L.) in Saxony-Anhalt, Germany. Fifteen Years Persistence to Date but No Spatial Dispersion

**DOI:** 10.3390/genes7010003

**Published:** 2016-01-16

**Authors:** Anke Belter

**Affiliations:** Saxony-Anhalt State Office for Environmental Protection, Reideburger Str. 47, 06116 Halle/Saale, Germany; anke.belter@lau.mlu.sachsen-anhalt.de; Tel.: +49-345-5240-654

**Keywords:** genetically modified (GM) plants, field trial, oilseed rape (OSR), *Brassica napus*, herbicide resistance, glufosinate, volunteers, persistence, dormancy, monitoring

## Abstract

Oilseed rape is known to persist in arable fields because of its ability to develop secondary seed dormancy in certain agronomic and environmental conditions. If conditions change, rapeseeds are able to germinate up to 10 years later to build volunteers in ensuing crops. Extrapolations of experimental data acted on the assumption of persistence periods for more than 20 years after last harvest of rapeseed. Genetically-modified oilseed rape—cultivated widely in Northern America since 1996—is assumed not to differ from its conventional form in this property. Here, experimental data are reported from official monitoring activities that verify these assumptions. At two former field trial sites in Saxony-Anhalt genetically-modified herbicide-resistant oilseed rape volunteers are found up to fifteen years after harvest. Nevertheless, spatial dispersion or establishment of GM plants outside of the field sites was not observed within this period.

## 1. Introduction

Oilseed rape (*Brassica napus*) is an important crop plant for cooking oil and biodiesel production, which in 2014 was cultivated worldwide in temperate regions on approximately 36 million hectares [1]. Genetically-modified OSR lines were first cultivated in 1996 and some 20 years later are found mostly in Canada (8 M ha), the USA (729,000 ha), and Australia (342,000 ha). This means 25% of worldwide OSR cultivation is GM (status as of: 2014) [1]. The main genetic modifications in OSR cultures relate to resistance against the broad-spectrum herbicides glyphosate (e.g., in *RoundUp*^®^, Monsanto, St. Louis, MO, USA and glufosinate (e.g., in *Liberty*^®^ and *Basta*^®^, Bayer Crop Science, Monheim, Germany. In Europe, some OSR lines genetically modified for herbicide resistance are approved with appropriate labeling for import, processing and use in human food and animal feed. Commercial cultivation of GM OSR, on the other hand, is not allowed in the EU [2].

Around the turn of the millennium, various field trials with genetically-modified OSR lines serving to test agronomic characteristics under field conditions were underway in Europe. In Germany, field trials with genetically-modified organisms (GMO) are subject to the *Gentechnikgesetz* (GenTG)—genetic engineering law, *i.e.*, authorization is to be obtained from the competent national authority, the German Federal Office for Consumer Protection and Food Safety (BVL) [3]. This office imposes conditions on the carrying out of the individual plans for deliberate release of GMO into the environment. In case of GM plants a deliberate release is only concluded once GM volunteers are no longer detected in the fields. The responsible surveillance authorities of the individual German states are to monitor compliance with the conditions of the corresponding releases into the environment within their territory. In the period from 1994 to 2007, more than 300 field trials with 15 different GM oilseed rape events took place at 88 different sites in Germany ([4] and information from the BVL on request). In particular, 247 fields at 62 different sites all over Germany were sown with glufosinate-resistant oilseed rape (events: GS 40/90 pHoe/Ac, Liberator C/6Ac and MS8/RF3).

One biological property of rapeseed that is sometimes problematic in farming is the high amount of seed losses during harvest by shedding. Losses up to 10% of the harvest are common, meaning often more than 10,000 seed·m^−2^ [5]. If shed seeds are brought into the deeper layers of soil through overturning tillage (plowing) shortly after the harvest, they can develop secondary seed dormancy in correspondingly unfavorable environmental conditions, like drought and darkness [6,7,8]. Numerous agronomic studies on the persistence of OSR in cropland proved the common property of rape seeds to remain viable in the soil seed bank there for many years [5,9,10,11,12,13,14,15]. OSR plants often are found as volunteers in following crops if the seeds are brought up again to the surface through field work [16,17,18]. Previous scientific studies on conventional OSR seeds proved a persistence capability of more than 10 years in the soil [7,9,10,19,20].

There have also been several publications of observations of the persistence of genetically-modified rapeseed in cropland and release sites. If one considers only the fields on which no rapeseed was deliberately cultivated between a GM OSR harvest and the respectively-described study, there is observation data e.g., for glyphosate-resistant canola in California over four years [14], for bromoxynil-resistant spring OSR in Canada over seven years [13], for various herbicide-resistant OSR varieties in France over up to eight years [5], as well as for PGS spring rape (events MS1/RF1, MS1/RF2, MS5/RF2) in Sweden over 10 years [12]. Lutman *et al.* reported in 2005 on their 30-month studies of seed persistence of herbicide-resistant GM OSR lines in Great Britain [15]. From their data, the authors extrapolated a period of 3–9 years for up to 95% degradation of the corresponding seed in the soil seed bank, and even 5–17 years for up to 99% degradation. Similar data were calculated by Middelhoff *et al.* [21].

From 1996 to 2002, a total of fifteen deliberate release trials with genetically-modified winter oilseed rape took place at eight sites in Saxony-Anhalt (Central Germany). Released were four different GM OSR events with herbicide resistance against glyphosate or glufosinate. Part of the glufosinate-tolerant rape lines (with *bar* gene for phosphinotricine acetyl transferase) were also F1 hybrids between a male sterile line and a fertility restorer line (*barnase* and *barstar* transgenes, see Table 1). For the individual releases into the environment, particular different conditions were applied to the approvals for the releasing companies (called operators) regarding the set-up of the field during trials (isolation distance, isolation track) and also with respect to the measures that were to be taken after the harvest (fallow period, irrigation if necessary, destruction of sprouting seedlings, avoidance of overturning tillage) [22]. The monitoring of compliance with these conditions is the task of the responsible federal state authority (up until 2002 it was the *Regierungspräsidium Magdeburg*, afterwards the Federal State Administration Office) of Saxony-Anhalt. Since 1999, the federal state authority have charged the laboratory of the State Office for Environmental Protection with the monitoring of field trial sites (FTSs) and their surroundings, as well as with the taking of samples and their testing for genetic modification.

In the following, the results of this official long-term surveillance of GM oilseed rape FTSs in Saxony-Anhalt are described.

**Table 1:** Deliberate release and follow-up checks of genetically-modified OSR in Saxony-Anhalt.

GM OSR Event (Abbreviation, Traits)	Release Site (BVL File Number of the Release Approval) [15]	Duration of Release (Years sown)	Operators Follow-Up (Number of Years)	Last Official Monitoring
**MS8/RF3:** glufosinate resistance (*bar*); male sterility (*barnase*) × fertility restorer (*barstar*)	Böhnshausen (6786-01-0090)	1999–2000 (2)	2001; 2002 (2)	2007
	Krüden (6786-01-0090)	1998 (1)	1999; 2000 (2)	2007
	Eickendorf (6786-01-0090)	2001 (1)	2002–2015 ^a^ (14)	2015
**GM OSR Event (Abbreviation, Traits)**	**Release Site (BVL File Number of the Release Approval) [4]**	**Duration of Release (Years sown)**	**Operators Follow-Up (Number of Years)**	**Last Official Monitoring**
**GS 40/90 and/or Liberator C/6Ac:** glufosinate resistance (*pat*)	Bottmersdorf (6786-01-0043, -0052 and -0053)	1996–1998 (3)	1999–2006 (8)	2008
	Böhnshausen (6786-01-0101)	1999–2000 (2)	2001; 2002 (2)	2007
	Rohrberg (6786-01-0101)	1999 (1)	2000; 2001 (2)	2007
	Etzdorf (6786-01-0101)	1999 (1)	2000–2015 ^a^ (16)	2015
**GT73:** glyphosate resistance (*epsps*)	Rahnsdorf (6786-01-0050)	1996–1997 (2)	1998–2002 (5)	2008
	Gerbitz (6786-01-0070)	1997–1998 (2)	1999–2003 (5)	2009
**MS1/RF1:** glufosinate resistance (*bar*); male sterility (*barnase*) × fertility restorer (*barstar*)	Etzdorf ^b^	1996–1998 (3)	none	2015

^a^ Follow-up checks not yet concluded; ^b^ No release, just Placing on the Market (cultivation) in accordance with Commission Decision No. 96/158/EG, Part C [23].

## 2. Materials and Methods

*Field Trial Sites (FTSs).* Each of the GM rape events released in Saxony-Anhalt is recorded in Table 1 giving the abbreviation and traits, as well as the corresponding site and duration of release. By referencing the listed BVL file number of the individual release approval, detailed information on the GM rape events can be called up in the BVL release database through the Web [4]. The measures actually undertaken in the fields during sowing and following harvesting are described in the interim and follow-up reports of the operators. The federal state authority was usually only briefly on site during sowing and harvesting operations; the multitude of individual farming activities could not always be checked immediately by the officials.

*Follow-up Checks by Operators.* The approvals provided for the corresponding FTSs to be controlled by the operating companies for escaped plants and volunteers several times a year after the harvest. If the operator noticed (GM) volunteers one year, the follow-up period was automatically extended by another year (see Table 1). During the entire period of the follow-up checks no rapeseed was allowed to be deliberately cultivated in these fields, the same applied to crops that could make follow-up checks for rape difficult (e.g., turnips (*Brassica rapa*) or mustard (*Brassica nigra*; *Sinapis alba*)).

*Cultivation of GM Winter Oilseed Rape.* In Etzdorf, on a trial site of the University of Halle-Wittenberg, MS1/RF1 rape (winter oilseed rape hybrid PGS-W3 with glufosinate resistance (*bar*) and male sterility (*barnase*)/fertility restorer (*barstar*)) from the company Plant Genetic Systems (PGS, Ghent, Belgium; since 2002 part of Bayer CropScience) was cultivated on a trial basis from 1997 until 2000. In contrast to deliberate releases into the environment (see above), this cultivation was a so-called *Placing on the Market* in accordance with Part C of the Commission Decision No. 96/158/EG, solely for the purpose of obtaining seed (not to be used for food or feed) [23]. There were no conditions attached to the approval for cultivation for any particular post-processing or follow-up on the part of the operator. In each of the three years of cultivation, another field on the trial site in Etzdorf was cultivated with MS1/RF1 rape. In 1998, the cropland of 0.5 ha was located just 50 m south of the area where GS-40/90 rape was released in 1999. The MS1/RF1 cultivation in 1999 (0.2 ha) was located 160m south of the simultaneous GS-40/90 rape release [24].

*Official Monitoring*. Official monitoring of the fields in which GM rapeseed was released or cultivated, was randomly carried out by the laboratory of the Saxony-Anhalt State Office for Environmental Protection in spring and/or fall; however, not every year at each location. The area of the former FTS was, if necessary, determined by GPS and systematically paced, usually by 2–3 persons. If only a few rape plants were found altogether, these were completely removed. If, however, at the time of sampling there was an accumulation of numerous rape seedlings (>1 per m^2^), up to 400 plants were sampled randomly, depending on the size of the field. All rape plants found at any time *outside* the former FTSs were always thoroughly sampled, e.g., on the field margins, on access roads and roadsides, on nearby woodpiles or ditches and in neighboring fields (as long as no conventional rapeseed was being cultivated there at the time).

*Plant DNA Extraction.* According to the number of rape plants found, these were pooled into composite samples of a minimum of three and a maximum of 20 pieces of leaf around 0.5 cm^2^ in size. Individual plants were also analyzed. For cell wall disruption, the plant material was either embrittled with the aid of liquid nitrogen and triturated with a micro pestle (Biozym Scientific GmbH, Hessisch Oldendorf, Germany, Art.-No. 710399) or crushed with ceramic beads (1.4 mm) in the sample homogenizer precellys24 (PEQLAB Biotechnologie GmbH; Erlangen, Germany). Extraction of genomic DNA was carried out over the years with various methods validated in the laboratory, which all led to equivalent DNA extracts: DNeasy^®^ Plant Mini Kit from QIAGEN (Hilden, Germany, Art.-No. 69104), NucleoSpin^®^ Plant Mini Kit from Macherey-Nagel (Düren, Germany, Art.-No. 740570.250), CTAB (Cetrimonium bromide) digestion (method of Tinker *et al.* [25]) and Maxwell^®^16 FFS Nucleic Acid Extraction Kit from Promega GmbH (Mannheim, Germany, Art.-No. X9431).

*Qualitative PCR*. Detection of genetic modification in the DNA extracts was carried out using qualitative construct-specific PCR screening procedures applicable to the released OSR events. These procedures were developed between 1998 and 2002 in the “Method Development” Committee of the German Federal/State Genetic Engineering Working Group (LAG) and validated by round-robin tests [26,27,28]. In particular was the development of the PCR detection methods for the pSSUAra-bar construct (in events MS8; RF3; MS1; RF1/2; see Table 2) that was initiated and coordinated by the author [29].

**Table 2:** Oligonucleotides for PCR screening on GM OSR.

Construct or/and Detectable GM OSR Events	Forward Primer (5′ → 3′)	Reverse Primer (5′ → 3′)	PCR Products (OSR Only)	Reference
**pSSUAra-bar construct** (MS8; RF3; MS1; RF1/2)	PGS-bar-A2: GAAGTT GACCGTGCTTGTCT	PGS-bar-B2: CAAGTC CACCAGGCAAGTAA	454 bp (MS8, RF3); 624 bp (MS1; RF1/2)	this work; [26,29]
**p35S-pat construct** (GS40-90; Liberator C/6Ac)	CaMV-F: ATCCTTC GCAAGACCCTTCCTC	pat 3-R: CCCAACC TTTGATGCCTATGTG	386 bp	[27]
**pFMV-epsps construct** (GT73)	CP1: GACTTACGAGCA GTTGCTGGACGGCTGC	pFMV2: CCTGACAGCCC ACTCACTAATGCGTATG	491 bp	[28]

For the studies described here, the HotStarTaq Master Mix Kit (Qiagen, Hilden, Germany, Art.-No. 203446) was always used, as well as the oligonucleotides (manufactured by TIB Molbiol, Berlin, Germany or by Eurogentec Deutschland GmbH, Cologne, Germany) listed in Table 2 and Table 3. The respective reactions and PCR conditions are reported [26,27,28,29,30,31,32,33,34,35]. All PCR reactions were performed with a “Mastercycler Gradient” thermocycler (Eppendorf AG, Hamburg, Germany). In our laboratory the following annealing temperatures were applied for screening PCRs (see Table 2): 1 min. 60 °C (all pSSUAra-bar constructs and p35S-pat construct) and 1 min. 63°C (pFMV-epsps construct). Positive analyses were further verified either with a second construct-specific primer pair or with event-specific detection (see Table 3; qualitative PCR only) [30,31,32,33,34,35]. Analysis of the PCR products was carried out by agarose gel electrophoresis in 1.5% agarose gels [36].

**Table 3:** Oligonucleotides for verification of positive screening PCR results.

Construct or/and Detectable GM OSR Events	Forward Primer (5′ → 3′)	Reverse Primer (5′ → 3′)	PCR Products (OSR Only)	Reference
**pSSUAra-bar construct** (MS8; RF3; MS1; RF1/2)	PGS-bar-A1: GTGCTT GTCTCGATGTAGTG	PGS-bar-B1: CGATAG GGAAGTGATGTAGG	1024 bp (MS8, RF3); 1194 bp (MS1;RF1/2)	this work *
**GS40-90: “Avalon” + “Falcon”** (first p35S-pat copy); event specific	Hess-443: TTACGGC GAGTTCTGTTAGGTCC	Hess-444: TGTTCACA TGAGACCATGCACG	266 bp (Avalon, Falcon)	[30]
**GS40-90: “Falcon”** (second p35S-pat copy); event specific	Hess-316: GCCAAGCT CAGGATCAGATTGTC	Hess-337: TTGTGGACG CTCTGGTGATAGTGC	210 bp (Falcon only)	[30]
**Liberator C/6Ac**; event specific	Hess-340: GATTGTC GTTTCCCGCCTTC	Hess-341: CGCAGGAGA GATACCGATAAGACTG	335 bp	[30]
**GT73**, event specific	RT73-1: CCATATTGAC CATCATACTCATTGCT	RT73-2: GCTTATACGA AGGCAAGAAAAGGA	108 bp	[31]
**MS1**, event specific	MLD025-F: ACGCTG CGGACATCTACATT	MDB175-R: CTAGATCG GAAGCTGAAGATGG	187 bp	[32]
**MS8**, event specific	KVM085: TTAGAAAAAGTA AACAATTAATATAGCCGG	HCA048: GGAGGGT GT TTTGGTTATC	129 bp	[33]
**RF1**, event specific	MDB118-F: CTAAGGG AGGTCAAGATGTAGC	KVM170-R: CGGGCC TAACTTTTGGTGTG	113 bp	[34]
**RF3**, event specific	KVM084: AGCATTTAG CATGTACCATCAGACA	DPA165: CATAAAGGA AGATGGAGACTTGAG	139 bp	[35]

* PCR conditions are the same as in [26].

## 3. Results

The mandatory follow-up checks of the FTSs by the operators were concluded in each of four cases (at three of the eight sites) already one year after harvest of the released genetically-modified rape, as no volunteers were detected at the site (see Table 1). In Krüden and Rohrberg, there was early termination of the trial (prior to flowering) at each site on account of the fields being affected by backwater due to bad weather. In Böhnshausen, the operator destroyed the entire crop of GS 40/90 and Liberator C/6Ac by chopping it up at the beginning of April (prior to seed maturation) because the trial had been revoked by the German Federal Office of Plant Varieties. The population of MS8/RF3 at the same site was swathed two weeks before harvesting to achieve simultaneous maturing of the pods in all trial plots. The harvest was followed by two months of only shallow cultivation with a roller or rotary harrow before the field was prepared and plowed for the subsequent crop, winter wheat. At the official monitoring of the Böhnshausen site in 2007, one of 200 volunteers found was genetically modified (MS8/RF3). This single find was within the former FTS and did not lead to a renewal of the operator’s duty to follow-up that had lapsed in 2002.

**Figure 1:**
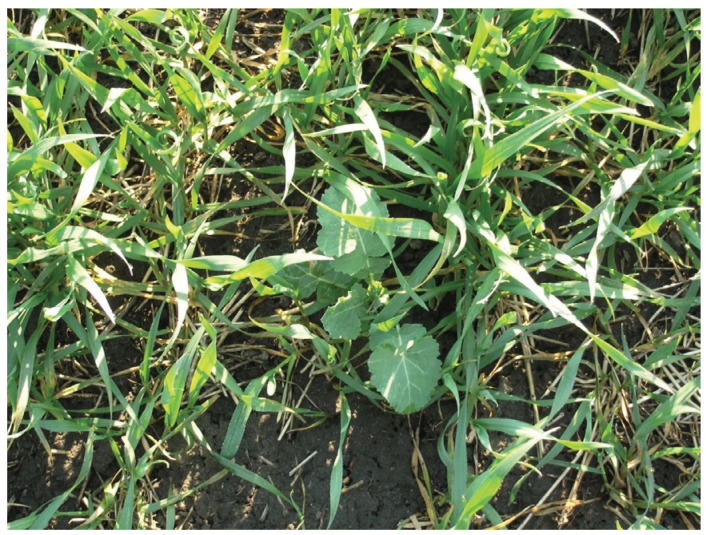
GM volunteer ORS (GT73) in a grain field near former FTS in Gerbitz, 2007. Picture shows a usual finding situation of OSR volunteers over the years at the former FTSs. In most of the cases volunteers could not grow up to build flowers and seeds because of different crop managements (Photo: Anke Belter).

At the Rahnsdorf, Gerbitz, and Bottmersdorf sites, the respective operators had to extend the follow-up checks by one year several times, since there was still volunteer rape growth detected four and seven years following harvesting (see Table 1). In 2007, during official monitoring at the Gerbitz site, a genetically-modified rape plant (GT73) was detected in a grain field directly adjacent to the former FTS. During official analysis of the harvested crop of conventional rape from this field the following year, the genetic modification (GT73) was not detected (detection limit of the test plan: 0.03%). This single find (Figure 1) did not lead to a renewal of the operator’s duty to follow-up that had lapsed in 2003.

At two of the eight former release sites in Saxony-Anhalt, the follow-up by the operator, as well as by the officials has not been concluded yet due to regular annual detection of GM rape (status as of October 2015, see Table 1). Results of the previous studies at both of these sites will be explained in more detail in the following.

### 3.1. Etzdorf (Steuden)

This is a relatively small FTS (0.33 ha) within a heavily structured agricultural experimental station belonging to the University of Halle-Wittenberg. In fall 1999, around 170,000 seeds were sown of glufosinate-resistant winter oilseed rape, “Avalon” transformation event (with one copy of the p35S-pat gene cassette) with a surrounding isolation track 3m in width separating it from a small frame of conventional rape (Artus variety). The harvest took place in July 2000 when the rape was fully ripe. A day later, the harvested seeds were destroyed in a hammer mill and subsequently spread on the trial site. After repeated shallow cultivation in the field and prior to preparing the seedbed for the subsequent crop of winter wheat, secondary tillage was carried out in order to destroy all rape plants that had sprouted in the meantime.

During the 2002/2003 growing season, conventional winter oilseed rape was cultivated and harvested on the former FTS by the Etzdorf experimental station guarantors, three years after the GS 40/90 deliberate release. This happened accidentally and contrary to the authority’s condition to keep the field free of rapeseed cultivation until the end of the follow-up. Through this incident, without a doubt, genetically-modified ORS GS 40/90 was grown again in this field and could have again led to crop loss of genetically-modified seed, although certainly to a much lesser extent than in the actual release. In the following years, this site was, therefore, meticulously monitored and examined for GM volunteers. From 2006 on, in addition to the GS 40/90 OSR, progeny of another genetically-modified rapeseed event (MS1/RF1) was occasionally found in the immediate vicinity. This originated from the cultivation of 1998, just 50 m away, that up until then had not been part of the official monitoring (see Table 1). Nevertheless, hybrid plants between GS 40/90 and MS1 or RF1 oilseed rape were not detected at any time. Furthermore, no genetically-modified rapeseed plants were detected in the vicinity of the former FTSs and cropland in Etzdorf (access roads, roadside, field margins) during the entire period.

The monitoring results of OSR samples taken in and directly adjacent to the former FTS at Etzdorf are summarized in Table 4.

**Table 4:** Official monitoring results on GM OSR volunteers at the Etzdorf site.

Year ^a^	Crop Rotations	Number of OSR Volunteers in and Directly Adjacent to the FTS		
		Total Number (in Brackets: Number of Generated Composite Samples)	GM OSR: GS 40/90 (Composite Samples)	GM OSR: MS1 and/or RF1 ^b^ (Composite Samples)
1999	Winter Barley	not studied		GMO grown in 1999
2000	GM winter OSR GS 40/90			GMO grown in 2000
2001	Winter Wheat	not studied ^c^		not studied
2002	No info available			
2003	Winter OSR			
2004	Winter Wheat	57 OSR plants (17 Composite samples)	11	
2005	Winter Barley	no OSR plants found ^c^	0 ^c^	
2006	Beans/Peas	44 OSR plants	6	1
2007	Winter Barley	41 OSR plants (10 Composite samples)	2	1
2008	Winter Wheat	25 OSR plants (1 Composite sample + 11 single plants)	4	1
2009	Winter Wheat	8 OSR plants	0 ^c^	1
2010	Durum Wheat	145 OSR plants (18 Composite samples)	7	0
2011	Beans/Peas/Soy	52 OSR plants (11 Composite samples)	6	0
2012	Ribbon Grass	174 OSR plants (17 Composite samples)	1	8
2013	Ribbon Grass	21 OSR plants ( 7 Composite samples)	1	0
2014	RG ^d^/WW ^e^	88 OSR plants (17 Composite samples)	5	0
2015	RG and others	185 OSR plants (22 Composite samples)	0 ^c^	7

^a^ Year of harvest; ^b^ No release, just Placing on the Market (cultivation) in accordance with Commission Decision No. 96/158/EG, Part C [23]; ^c^ Detection of (GM) OSR volunteers in the field by the operator only during his mandatory follow-up; ^d^ Ribbon Grass; ^e^ Winter Wheat.

### 3.2. Eickendorf

In contrast to Etzdorf, the Eickendorf site is a ten-fold larger release site (3.75 ha) located within a very open and level agrarian landscape that, because of the fertile loess-chernozem soil, has been dominated by large-scale farming for decades. In 1934, as part of the so-called Reich’s Soil Appraisal, the soil existing here was awarded a soil value number of 100 (highest rating) because of its natural yield conditions [37]. Since the end of World War II, no rapeseed had ever been cultivated here.

In fall 2001, 1.83M seeds of MS8/RF3 winter oilseed rape (glufosinate resistance (*bar* gene) and male sterility (*barnase*)/fertility restorer (*barstar*)) were sown, using the so-called field-in-field technique. The GMO release site (diverse varieties with the same genetic modification in narrow plots and multiple repetitions in parallel), surrounded by a 10 m-wide uncultivated arable strip, was located in the midst of a significantly larger conventional OSR field (Mohican variety). In June 2002, during the deliberate release, our laboratory took green rape pods from the conventional rape field adjacent to the GMO site (distance: 10m uncultivated arable strip) and analyzed the green rape seeds for genetic modification. As expected, outcrossing events from the FTS were detected in the surrounding field. In a similar sample taken at a distance of 200 m from the FTS, no MS8/RF3 was detected (data not shown). Following the harvest at the end of July 2002, the genetically-modified harvested crop from Eickendorf was transported by the operator to a company site in Mecklenburg-Vorpommern, where it was destroyed and spread on a similar release site (also there). A 6 m-wide strip of the conventional rape field surrounding the release site in Eickendorf was harvested separately and processed into industrial oil in a nearby oil mill. The rest of the harvested crop of the surrounding rape field (78.32 t) was processed to biodiesel. As per the operator’s report the harvested field was harrowed (shallow cultivation) twice before the subsequent crop, winter wheat, was drilled without plowing at the beginning of October 2002. In the subsequent follow-up of the former FTS by the operator and officials, the margins of the entire field were included at regular intervals. GM OSR plants have not been detected in the vicinity, neither on the field margins nor on access roads or roadsides since 2004. Only in 2006, a single GMO plant (MS8/RF3) was detected within the field (on the arable land) in a provisional access track 100 m north of the former FTS.

The monitoring results of the OSR samples taken in and directly adjacent to the former FTS at the Eickendorf site are summarized in Table 5.

**Table 5:** Official monitoring results on GM OSR volunteers at the Eickendorf site.

Year ^a^	Crop Rotations	Number of OSR Volunteers in and Directly Adjacent to the FTS	
		Total Number (in Brackets: Number of Generated Composite Samples)	GM OSR: MS8 and/or RF3 (Composite Samples)
before	*no OSR since 1945*	not studied	not studied
2001	Winter Wheat		
2002	GM winter OSR MS8/RF3	*Analyses on the range of GMO outcrossing into the adjacent conventional OSR field (data not shown)*	
2003	Winter Wheat	no OSR plants found ^b^	0 ^b^
2004	Winter Wheat	no OSR plants found ^b^	0 ^b^
2005	Potatoes	55 OSR plants (4 Composite samples)	4
2006	Winter Wheat	18 OSR plants	4
2007	Maize	no OSR plants found ^b^	0^b^
2008	Facultative Wheat	no OSR plants found ^b^	0^b^
2009	Winter Wheat	236 OSR plants ^c^ (25 Composite samples)	0^b^
2010	Potatoes	1 OSR plant	0^b^
2011	Winter Wheat	395 OSR plants (39 Composite samples)	15
2012	Winter Wheat	84 OSR plants (11 Composite samples)	6
2013	Maize	18 stem rests and 13 OSR plants (2 Composite samples)	0 ^b^
2014	Winter Wheat	25 OSR plants (5 Composite samples)	3
2015	Potatoes	121 OSR plants (19 Composite samples)	6

^a^ Year of harvest; ^b^ Detection of (GM) OSR volunteers in the field by the operator only during his mandatory follow-up; ^c^ Sampling along the verge of an adjacent field (distance to FTS: 80 m).

The number of volunteer OSR plants found in Etzdorf und Eickendorf varied greatly from year to year. This is due to the prevailing conditions at the respective sites at the time of sampling, particularly to the status of the field preparation at the time and general weather. Common agricultural use took place on these former field trial sites, except cultivation of OSR. Official monitoring was carried out usually only once a year, in rare cases twice (e.g., in fall, if the field was freshly prepared in spring). The target of the monitoring was always purely for the detection of GM plants (even just a single one), not for documenting a trend.

## 4. Discussion

The monitoring results prove experimentally for the first time the persistence of genetically-modified oilseed rape in arable fields over 13 and 15 years after their (one-time) agronomic testing. At two of the eight former release sites in Saxony-Anhalt, the follow-ups are still not concluded (status as of October 2015) while at six sites no GM volunteers have been detected for several years.

What are the reasons for this difference?

Apart from the trial terminations prior to flowering or seed maturation in Krüden, Rohrberg, and Böhnshausen, the different soil conditions are to be discussed first. The field trials in Saxony-Anhalt represent soil types of varying quality: from poor sandy soil (Rahnsdorf) to sandy clay (Gerbitz), clay soil (Krüden, Rohrberg), and primarily loess (Böhnshausen) up to loess-chernozem soil in Bottmersdorf, Etzdorf, and Eickendorf. It becomes apparent that in the most fertile soil the persistence of the rape seeds is most pronounced, namely at the last three of the sites mentioned. This is consistent with studies by Gruber *et al.* showing that soil with good water retention capability favors the survival of escaped rape seeds [38].

The most important source of OSR volunteers in ensuing crops is seed loss by shedding during OSR harvesting, followed by plowing to allow seeds to enter the soil seed bank and develop secondary dormancy. However, there was certainly less influence from the extent of seed loss during harvest on the FTSs, since all fields (except the terminated trials) were harvested only when the seeds were fully ripe, so that in this respect comparable conditions at the sites can be assumed. The same can be assumed for tillage after harvesting until sowing of subsequent crops, since according to operator reports care was taken at all sites during a harvest year not to do any overturning, just shallow cultivation. Additionally, escaped rapeseeds from the harvest was sprouted at all sites (at some, several times) and then destroyed. Nevertheless, a number of GM seeds must have reached the soil seed bank and developed secondary dormancy. Reliable information on the specific weather conditions at the time of harvest at the respective sites is unfortunately not available and, thus, cannot be part of this discussion. The same applies for the influence of variety-specific traits on persistency, since at the various sites, genetically-modified lines of different varieties of OSR were used, in Eickendorf even diverse varieties with the same genetic modification in narrow plots and multiple repetitions in parallel. In Etzdorf, the accidental cultivation of conventional OSR three years after the GS 40/90 deliberate release certainly had an impact on the total content of GM rape seeds in the soil seed bank and thus on volunteers in the following years.

Of particular interest are the continuing findings of MS1/RF1 rape in Etzdorf 15 years after its cultivation on a relatively small field (0.5 ha). For the operator, this cultivation was not connected with any of the usual conditions later applied to areas of deliberate releases of GMO regarding post-processing and follow-up checks, such as no tillage with a plow after harvesting or avoidance of OSR as a subsequent crop. In direct comparison with the neighboring FTS of GS 40/90 rape, one comes to the conclusion that the missing security measures probably had less to do with the survival of the rape seeds in the soil than the special site conditions in Etzdorf, such as soil quality.

A more sobering result of this monitoring is that with ongoing agricultural use there appears to be no single agronomic measure suitable for keeping fields free of OSR seeds and volunteers—with or without genetic modification—once and for all. The found GM OSR volunteers must be either seedlings from original released GM seeds or direct progeny of them. For instance we detected both, hybrid plants (MS8 × RF3) and single events (MS8 and RF3) in Eickendorf 2015, the latter due to crossing events with conventional OSR plants in neighboring non GM plots or frameseed during the deliberate release years ago. In this respect, the trait of persistence in the soil seed bank proves the correctness of the thesis of the so-called “substantial equivalence” of conventional and genetically-modified OSR, except the trait [39,40,41].

Not least, observations over the entire monitoring period of 15 years show that based on the former FTSs and cropland there was no spatial dispersal of GM OSR detectable in the surrounding area, much less a stable establishment in the environment. However, since this was usually only a one-year cultivation (occasionally up to three years in a row, see Table 1) it was not even assumed (*cf.* [42]). However, it should be considered that in Etzdorf and Eickendorf different herbicides (also glufosinate, which could serve as a selector in this case) were routinely used to control weeds at tracks and fields adjacent to the former FTSs. Our own sampling and analyses of feral rapeseed plants along railway lines in the big cities Magdeburg and Halle in 2010 and around an oilseed mill in Magdeburg resulted in no genetically-modified OSR detection (data not shown).

More or less permanent population formation of feral *Brassica napus* (conventional and GM) on ruderal areas and transport paths was always reported as being only in connection with multiple rapeseed cultivations in a field or region and/or spillage during the regular transport from the harvest site or import site (e.g., ports) to the processing or export site (oil mills, ports). Yoshimura *et al.* [43] and Knispel *et al.* [44] documented feral GM OSR populations in western Canadian landscapes (Saskatchewan, southern Manitoba, port of Vancouver) where genetically-modified canola has been continuously large-scale cultivated and transported since 1995. Saji *et al.* [45], Aono *et al.* [46], Kawata *et al.* [47], and Nishizawa *et al.* [48] described dispersion and persistence of genetically-modified oilseed rape around Japanese ports and roadsides while no GM OSR was cultivated in Japan to date. However, GM OSR was imported by Japan for oil production for years. The question “Where do the feral oilseed rape populations come from?” is answered amongst others by Pessel *et al.* [49] and Pivard *et al.* [50] in France, von der Lippe *et al.* [51] in Germany (Berlin city), and Schafer *et al.* [52] in the United States (North Dakota). Notable in this context are also observations by Schulze *et al.* (2014) about unexpected diversity of feral GM OSR despite a cultivation and import ban in Switzerland [53]. Swiss authors detected GT73 and MS8/RF3 OSR plants in a Rhine port, where they built small populations due to losses from a shipload of Canadian wheat contaminated with rapeseed. Nevertheless the presence of feral rapeseed populations is discussed to be not an environmental problem itself, and even GM herbicide-resistant OSR has not become invasive outside cultivated and ruderal habitats to date [54,55].

## 5. Conclusions

The monitoring results reported here allow following conclusions: Long-term persistence of viable rape seeds at agronomic sites is exceedingly promoted by high quality soil conditions. It is very difficult to remove rape seeds that once reached the soil seed bank and hinder them to germinate years later. Our observations showed no spatial dispersion effects of genetically-modified herbicide resistant OSR in the environment of the former release sites over many years despite of the persistence. All these findings are able to prove the substantial equivalence of conventional and GM OSR, except the trait. 

In Saxony-Anhalt, official monitoring for genetically-modified OSR volunteers is to be continued in the years to come at the former field trial sites in Etzdorf and Eickendorf and their environment, to securely prove experimentally for future risk assessment its maximum capacity for persistence.
